# CYP450 phenotyping and accurate mass identification of metabolites of the 8-aminoquinoline, anti-malarial drug primaquine

**DOI:** 10.1186/1475-2875-11-259

**Published:** 2012-08-02

**Authors:** Brandon S Pybus, Jason C Sousa, Xiannu Jin, James A Ferguson, Robert E Christian, Rebecca Barnhart, Chau Vuong, Richard J Sciotti, Gregory A Reichard, Michael P Kozar, Larry A Walker, Colin Ohrt, Victor Melendez

**Affiliations:** 1 Walter Reed Army Institute of Research, 503 Robert Grant Ave, Silver Spring, MD, 20910, USA; 2 AB Sciex, 500 Old Connecticut Path, Framingham, MA, 01701, USA; 3 National Center for Natural Products Research, School of Pharmacy, University of Mississippi, Oxford, MS, 38677, USA

## Abstract

**Background:**

The 8-aminoquinoline (8AQ) drug primaquine (PQ) is currently the only approved drug effective against the persistent liver stage of the hypnozoite forming strains *Plasmodium vivax* and *Plasmodium ovale* as well as Stage V gametocytes of *Plasmodium falciparum*. To date, several groups have investigated the toxicity observed in the 8AQ class, however, exact mechanisms and/or metabolic species responsible for PQ’s haemotoxic and anti-malarial properties are not fully understood.

**Methods:**

In the present study, the metabolism of PQ was evaluated using *in vitro* recombinant metabolic enzymes from the cytochrome P450 (CYP) and mono-amine oxidase (MAO) families. Based on this information, metabolite identification experiments were performed using nominal and accurate mass measurements.

**Results:**

Relative activity factor (RAF)-weighted intrinsic clearance values show the relative role of each enzyme to be MAO-A, 2C19, 3A4, and 2D6, with 76.1, 17.0, 5.2, and 1.7% contributions to PQ metabolism, respectively. CYP 2D6 was shown to produce at least six different oxidative metabolites along with demethylations, while MAO-A products derived from the PQ aldehyde, a pre-cursor to carboxy PQ. CYPs 2C19 and 3A4 produced only trace levels of hydroxylated species.

**Conclusions:**

As a result of this work, CYP 2D6 and MAO-A have been implicated as the key enzymes associated with PQ metabolism, and metabolites previously identified as potentially playing a role in efficacy and haemolytic toxicity have been attributed to production via CYP 2D6 mediated pathways.

## Background

Despite encouraging levels of progress in international control efforts, as many as 3.3 billion people in the world are at continued risk for malaria infection [1]. While *Plasmodium falciparum* exacts a greater burden in mortality and morbidity, the impact of *Plasmodium vivax* is also significant. It has been estimated that 40% of the world’s population are at risk of vivax malaria and, on the whole, more people are at risk of vivax than falciparum malaria [2,3].

A key component of continued control and eradication efforts is the development of effective drugs for treatment and prophylaxis. Only the 8-aminoquinoline (8-AQ) class of compounds have demonstrated the ability to target the key survival stages of the parasite — the sleeping liver stages, or hypnozoites, of *P. vivax* and *Plasmodium ovale*, and Stage V gametocytes of *P. falciparum*, with primaquine (PQ) being the only drug from this class in clinical use. Unfortunately, the use of 8-AQs is limited by their tendency to cause haemolytic anaemia in individuals with a genetic deficiency in glucose-6-phosphate dehydrogenase (G6PD), an enzyme implicated in the body’s defence against oxidative stress [4].

Although in use for several decades, PQ’s mechanisms of efficacy and toxicity are not well understood and its metabolic profile has not been fully elucidated. These mechanisms of efficacy/toxicity are believed to involve the formation of reactive oxygen species or interference by PQ and/or its metabolite(s) with electron transport in the parasite [5]. Further, it is generally believed that PQ’s haemolytic toxicity is due to one or more metabolites and not the parent compound [6]. For example, Link *et al.* show direct methaemoglobin (MHb) formation in canine hemolysates and purified human oxyhaemoglobin (Hb) upon exposure to the putative PQ metabolite 5-hydroxyprimaquine (5-HPQ) [7,8]. Further, Ganesan *et al.* have recently demonstrated, in a human erythrocyte-based model of PQ toxicity, the ability of several CYPs, most notably 3A4, 2D6, and 2B6, to form reactive oxygen species resulting in generation of methaemoglobin [9]. MAOs have also been implicated in PQ metabolism and may play a role in carboxyprimaquine (CPQ) formation [10].

Elucidation of the fundamentals of PQ metabolism, including the number, type, and relative contribution of involved metabolic enzymes, as well as the metabolites produced in each pathway, will help to determine if the drug’s efficacy is inextricably linked to its toxicity in G6PD-deficient individuals, or if future drug design efforts could overcome toxicity, and potentially enhance efficacy, by directing metabolism. To this end, a phenotyping study using the relative activity factor (RAF) method of Crespi *et al.* was completed to determine the number and type of mixed function oxidases involved in PQ metabolism and the relative contribution of each [11]. Overlap, if any, between the CYPs implicated in toxicity by Ganesan *et al.* and any CYPs shown to metabolize PQ to a great extent could help direct future metabolism studies toward ultimately resolving the question of which enzyme and which metabolite(s) is ultimately responsible for efficacy and observed haemolytic effects [9].

## Methods

### Chemicals

Chemicals used were: primaquine diphosphate (Sigma, # 160393), nicotinamide adenine dinucleotide phosphate, oxidized form (NADP) (Sigma, # 077K7000), acetonitrile (Fisher Scientific, # 972970), glucose-6-phosphate (G6P) (Sigma, # 046K3779), glucose-6-phosphate dehydrogenase (G6PD) (Sigma, # 068K3795), and magnesium chloride (MgCl_2_) (Sigma, # 102K0154). The mobile phase for LC separations consisted of acetonitrile, distilled water and formic acid; all were of HPLC-grade.

### Isoenzymes

Baculovirus-insect cell expressed recombinant Cytochrome-P450 Supersomes® containing CYP-1A2 (BD Biosciences, Billerica, MA, USA, # 18682), 2D6 (BD, # 16902), 3A4 (BD, # 24175), 2C9 (BD, # 25315), 2C19 (BD, # 15466), MAO-A (BD, # 31000), MAO-B (BD, # 36152), were stored at −80°C until required and were rapidly thawed by submersion in a 37°C water bath before use.

### Enzyme activity screening

Cofactor concentrations were as follows in all experiments: 1 U/ml G6PD, NADP 1.3 mM, G6P and MgCl_2_ 3.3 mM, and 0.5 mg/mL CYP, MAO-A, MAO-B in 0.1 mM pH 7.4 phosphate buffered saline (PBS). Final reaction volume was 1 mL. Reaction mixtures containing all cofactors and enzymes were pre-warmed for 10 min at 37°C, and reactions were started with the addition of primaquine. Aliquots of 250 μL were quenched after 2 h with an equal volume of cold acetonitrile. The resulting samples were centrifuged at 13,000 rpm for 10 min and the supernatant collected for analysis. Each experiment was conducted with an n of four to eight. Error was represented as the standard deviation.

### Primaquine analysis

LC-MS analysis for phenotyping studies and enzyme activity were performed using a Flux Rheos 2000 pump (Flux Instruments AG, Switzerland) coupled with a CTC PAL autosampler (LEAP Technologies, Carrboro, NC, USA) and a Micromass ZQ (now Waters Corp., Milford, MA, USA) mass spectrometer. Initial gradient conditions were 98% water and 2% acetonitrile, each with 0.1% formic acid. Organic content was raised from 2% to 40% over 3 min before returning to the initial conditions for equilibration for subsequent injections. The MS method detected primaquine (parent ion) by positive ion electrospray ionization at a m/z of 259.88. Analytical separations were achieved using a Waters X-Terra RP 5 cm x 2 mm, 3 μm C18 column, with a flow rate of 300 ml/min.

### RAF determination

Sample stocks of testosterone (3A4) (Sigma, # 160393), bufuralol (2D6) (Sigma, # UC168), S-mephenytoin (2 C19) (Sigma, # UC175), and serotonin (MAO-A) (Sigma, # H9523) at 10 mM in DMSO were diluted to a final concentration of 1 μM into a mixture containing 0.5 mg/mL of pre-incubated pooled human liver microsomes (BD Biosciences, Billerica, MA, USA) or recombinant enzyme of interest, 1.3 mM NADP, 3.3 mM MgCl_2_, and 0.1 M pH 7.4 PBS using a TECAN Genesis RSP 150 (Tecan, Durham, NC, USA) robotic liquid handler. The reaction was started with the addition of 1 U/mL G6PD. The mixture was incubated on a shaking platform at 37°C, and aliquots were taken and quenched with the addition of an equal volume of cold acetonitrile at 0, 10, 20, 30 and 60 min. Samples were centrifuged at 3,700 rpm for 10 min at 20°C to remove debris. Sample quantification was carried out by LC/MS. RAF was calculated using the equation:

(1) $$\text{RAF =}\;\left(\text{rate for probe substrate in HLM}\right)\;\text{÷}\;\left(\text{rate for a probe substrate with recombinant isoenzyme}\right)$$

[11].

### Kinetic studies

Incubations containing cofactors, primaquine (ranging from 0 μM to 40 μM), and enzyme (CYP, MAO-A, MAO-B) were treated as above, but quenched after 30 min (to remain within linear range of activity) with an equal volume of cold acetonitrile. Each experiment was conducted with an n of four to eight. Experimental data were fit to the Michaelis-Menten equation using non-linear least squares approximation. Error was represented as the standard deviation.

### Hepatocyte incubations

Pooled human hepatocytes and culture medium were purchased from Celsis/IVT (Chicago, IL). Cultures in suspension were initiated from cryopreserved vials using InVitro HI incubation buffer. Cell viability was verified under a microscope by trypan blue exclusion. Approximately 5.0x10^5^/ml hepatocytes were incubated with PQ for 2 h. Reactions quenched using two volumes cold ACN.

### Metabolite identification

All isoenzyme incubations were performed as mentioned in the enzyme activity screening section above, except PQ was fixed at 50 μM to increase the chances of detecting low-level metabolites, and incubations were quenched after 1 hr. Hepatocyte incubations were performed as outlined in the hepatocytes incubations section. Metabolites in the accurate mass data were found using AB Sciex MetabolitePilot™ software. The data were searched using an algorithm that used all of the following criteria for finding potential metabolites: predicted metabolites, mass defects, and isotope pattern (all three for typical phase I and phase II and combinations with dealkylations), and also common fragment ions and neutral losses found in the primaquine product ion spectrum. The data were scored by four criteria with user-selectable weighting: mass defect (20% of total) with an all-or-nothing score; isotope pattern (10% of total); MS/MS similarity and quality (30% of total); and mass accuracy (40% of total). The last three scoring criteria were given weighted values based on how close the measure property matched theory. Most metabolites had a score of 70% or better. The software allows for up to five controls. The data were processed using three controls (a time 0 sample, and two buffer blanks).

MetabolitePilot™ software also was used to help match probable structures to the MS/MS spectra of potential metabolites using its interpretation module. Using the accurate mass (typically within ca. 1 mDa except for very low m/z fragments) of the fragment ions and a program which allows for bond breakages, ring closures, and re-arrangements while also using chemical logic, the program presents possible structures for the fragments, highlighting them within the proposed structure.

## Results

### Metabolic enzyme phenotyping

**Figure 1:**
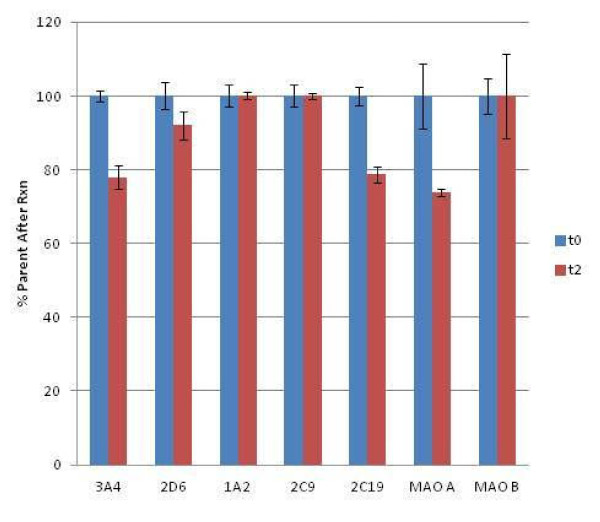
**CYP, MAO-A & B activity plotted as % parent loss for PQ after two-hour incubation.** Concentrations were as follows: PQ was at 10 mM, CYPs, MAOs were at 100 mg/ml, NADP was 1.3 mM, MgCl_2_ and G6P were both 3.3 mM, and G6PD was 1 Unit/ml. 3A4, 2D6, 2 C19, and MAO-A show activity. Each experiment was done with an n of four to eight. Error reported as SD.

**Figure 2:**
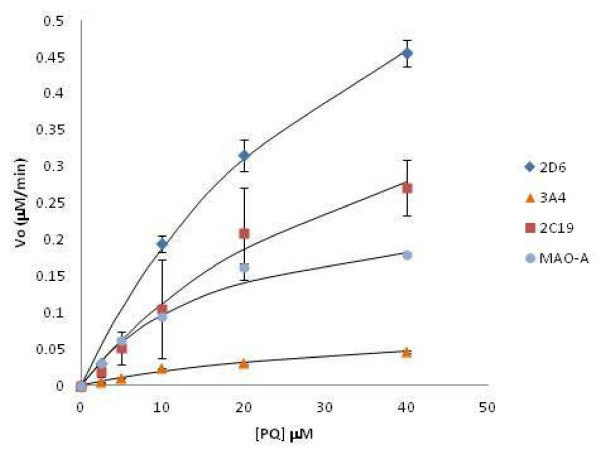
**CYPs, MAO-A steady-state kinetics with PQ. Intitial velocity (Vo) vs. PQ concentration.** Concentrations were as follows: PQ was from 0 to 40 mM, CYPs, MAOs were at 100 mg/ml, NADP was 1.3 mM, MgCl_2_ and G6P were both 3.3 mM, and G6PD was 1 Unit/ml. Reaction mixture was incubated for 30 min and quenched with cold ACN. Each experiment was done with an n of four to eight. Error reported as SD.

A panel of recombinant human metabolic enzymes (CYPs and MAOs) was screened for activity against 10 μM PQ. To account for drift in signal and spontaneous parent loss, each time point was compared to a PQ only (no enzyme present) sample incubated under the same conditions. After two-hour incubations, only 2C19, 2D6, 3A4, and MAO-A showed significant activity as measured by the loss of PQ (10 to 30%) (Figure 1). Each of the four enzymes that demonstrated activity against PQ was subjected to a steady-state kinetic analysis to determine K_m_ and V_max_, as reported in Table 1 and illustrated in Figure 2. As defined by Crespi *et al.*., calculation of the relative activity factor (RAF) from kinetic constants derived from cDNA expressed isoenzymes allows for a determination of individual contribution from each CYP to intrinsic clearance [11]. Briefly, each V_max_/K_m_ was weighted in this method by an experimentally determined constant (RAF) to account for the relative abundance of each CYP as expressed in microsomes, as well as the relative activities of each isoenzyme preparation. RAF is calculated as (rate for a probe substrate parent loss in HLM)/(rate of probe substrate parent loss with the cDNA expressed isoenzyme). RAF and a %-weighted contribution from each of the enzymes tested are found in Table 1. Weighting contributions by RAF normalizes for activity of cDNA expressed isoenzymes both for activity in the HLM component mixture and relative abundance of each of the component isoenzymes in HLMs only. Many caveats exist in the interpretation and extrapolation to *in vivo* systems, and these parameters should only be used as an indication of gross contribution to phase I metabolism.

**Table 1:** Steady-State kinetic parameters and RAF weighted contributions for PQ metabolism

	**K**_**m**_**(mM)**	**V**_**max**_^***a***^	**V**_**max**_**/K**_**m**_^*b*^	**RAF**^*c*^	**Contribution**^*d*^	**% Contribution**^*e*^
2D6	37 ± 3	35.2 ± 2.5	951.4	0.014	13.3	1.7
3A4	37 ± 2	3.6 ± 0.2	97.3	0.42	40.9	5.2
2 C19	52 ± 19	25.6 ± 9.5	492.3	0.24	118.2	17.0
MAO-A	17 ± 1	10.4 ± 0.5	615.4	0.97	596.9	76.1

^*a*^ Values in picomoles per minute per picomole of enzyme.

^*b*^ Values in nL/min/picomole of enzyme.

^*c*^ RAF = (rate for probe subs. with HLM)/(rate for probe subs. with isoenzyme).

^*d*^Contribution_i_ = (rate of metabolism for unknown with isoenzyme).

^*e*^% Contribution_i_ = (Contribution_i_/(∑ Contribution values))*100.

### Metabolite identification

While parent loss is an inherently less-sensitive means of monitoring metabolic activity compared to metabolite formation, it was chosen for these studies for two primary reasons. First, without *a priori* knowledge of which metabolites would be observed and, therefore, with no neat standards of the metabolites, it would be difficult to develop appropriate analytical methods to monitor their formation. Second, since all metabolites will presumably have unique ionization characteristics compared to other metabolites as well as the parent compound, no quantitative conclusions could be made about the amount of metabolite being formed viz. the absolute metabolic activity of PQ. Consequently, it was decided that loss of parent would be the most consistent metric across all enzymes. This second point should also be kept in mind in the following discussions of total chromatographic peak area. Peak area is a function of a compound’s ionization efficiency under the conditions of the analysis, and have not been calibrated or quantified in this study. The goal of this part of the study was to explore the variety and the nature of the metabolites being formed by the active enzymes, with the object being the differentiation and prioritization of the key metabolic pathways for further investigations towards an improved therapeutic index for PQ and other 8AQs. While unstable intermediates may exist and may have been undetected using the experimental methods described herein, it is asserted by the authors that the observed metabolites provide an adequate foundation for that stated purpose.

Preliminary nominal mass PQ metabolite identification experiments were performed using recombinant human CYPs 2D6, 3A4, and 2C19 and MAO-A and 50 μM PQ. These higher concentrations of PQ as compared to the phenotyping experiment were designed to increase the chances of seeing low level metabolites. The data showed a marked loss of parent with only MAO-A and 2D6 over the course of one-hour incubation period, while 3A4 and 2 C19 left PQ largely intact with only the formation of trace levels of oxidated and demethylated metabolites. The major observed metabolite in MAO-A incubations was consistent with de-amination and alcohol formation, while trace amounts of CPQ formation were also observed (Table 2). CYP 2D6 incubations led primarily to the formation of two isobaric metabolites with an MS/MS fragmentation pattern consistent with hydroxylation on the quinoline core (Table 2).

**Table 2:** **Putative metabolites observed after one-hour incubation with recombinant human MAO-A, 2D6, 3A4, and 2 C19 with retention times (rt), observed*m/z*and transitions**

**Putative metabolite**	**rt (min)**	***m/z*(transition)**	**MAO-A**	**2D6**	**3A4**	**2 C19**
**Parent**	6.98	260.3 → 175.1	60	80	99	98
**PQ-alcohol**	8.2	261.3 → 175.1	38			
**CPQ**	8.4	275.3 → 175.1	<1			
**Ox1 (quinoline core)**	5.0	276.3 → 191.1		5		
**Ox2 (quinoline core)**	6.1	276.3 → 191.1		6		

Tabulated data represents % of the total chromatographic peak area for each component. These values provide a qualitative relative measure of the metabolites produced in these incubations and should not be construed in any way as quantitative measurements.

**Figure 3:**
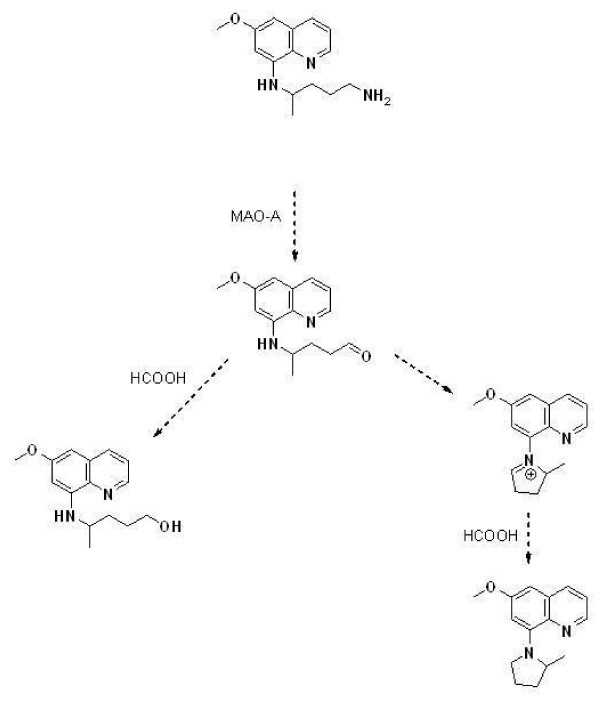
**Putative pathway to observed MAO-A mediated metabolites.** The PQ alcohol (*m/z* 261.2) is formed via formic acid mediated reduction of the aldehyde, whereas the *m/z* 243.1 is formed by aldehyde ring closure followed by formic acid reduction.

**Figure 4:**
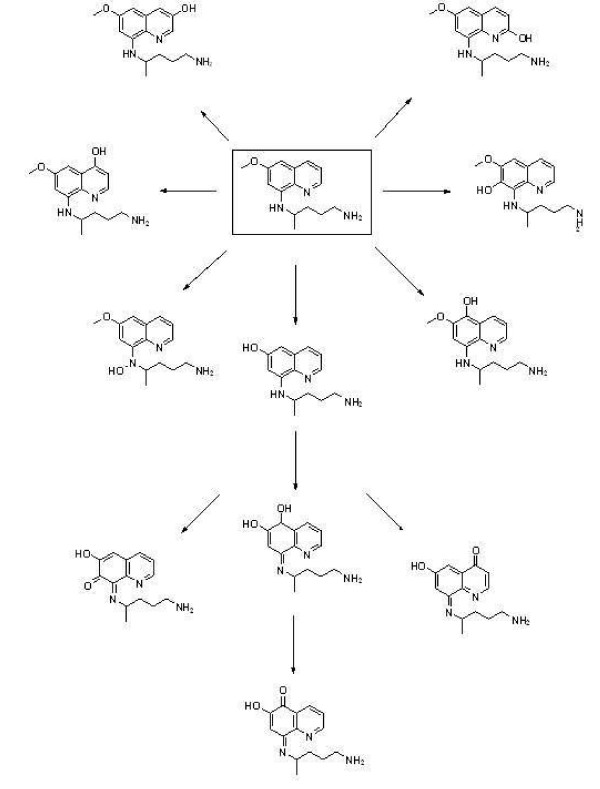
**Putative pathway to observed CYP 2D6 mediated phenolic and quinone metabolites.** Positional isomers on the quinoline core have different observed retention times, but are isobaric; therefore individual peaks cannot be assigned for the six observed phenols.

In order to confirm the identity of the metabolites observed using nominal mass instrumentation, duplicate preparations of the MAO-A and CYP 2D6 incubations were analysed using an AB Sciex TripleTOF™ 5600 mass spectrometer with high resolution accurate mass capabilities. Samples were analysed using a MDF IDA (on-the-fly, mass defect, filtered information-dependant acquisition) method in which the system gives preference to ions whose m/z ratio matches the expected mass defects of primaquine and its metabolites for the generation of MS/MS spectra for confirmation. Observations are summarized in Table 3. In short, after incubation with MAO-A, the alcohol was observed as in nominal mass experiments. In addition, a second *m/z* consistent with a ring-closed form of the expected aldehyde was also observed. As outlined in Figure 3, it was proposed that the observed alcohol is the by-product of reduction of the aldehyde by formic acid present in the chromatographic mobile phase. Incubations with 2D6 produced a variety of low level (<10% chromatographic peak area) phenolic, quinone, and demethylated metabolites (Figure 4). While recombinant enzyme incubations are a useful tool for correlating metabolites with their pathways of formation, they are not sufficiently predictive of the competitive processes in the liver in *in vivo* circumstances. Consequently, metabolite identification studies were also performed following *in vitro* hepatocyte incubations. Metabolites formed after two-hour incubations of PQ with pooled human hepatocytes were identified with accurate mass measurements and are listed in Table 4. Comparison with recombinant enzyme studies shows that of the 11 unique metabolites observed in hepatocytes, five could be directly attributed to the pathways outlined for CYP 2D6 and MAO-A. Namely, three phenolic metabolites were observed in hepatocytes, which had masses and retention times identical to ones observed after incubation with 2D6. The summed chromatographic peak areas for these phenolic metabolites accounted for less than 0.5% of the total. The PQ alcohol also appeared as observed after incubation with MAO-A, and, to a much lesser extent, CYP 2D6. The total chromatographic peak area for this metabolite was 0.3%. A demethylation, identified as occurring at the 6-methoxy position was also observed. It was identified as deriving primarily from the CYP 2D6 pathway and accounted for 0.1% of the total peak area. The largest single metabolite observed was CPQ, at 7.4% of the total chromatographic peak area. Three glucoronide conjugates were also observed along with two, as-of-yet, unidentified metabolites. Including CPQ as a presumptive by-product of aldehyde dehydrogenation, metabolites directly attributable to MAO-A and CYP 2D6-mediated pathways accounted for more than 93% of the total metabolite area observed after two-hour incubation with hepatocytes.

**Table 3:** **Putative metabolites observed using accurate mass after one-hour incubation with recombinant human MAO-A, 2D6 with retention times (rt), observed*m/z*and transitions**

**Putative Metabolite (2D6)**	**Formula**	***m/z***	**ppm**	**rt (min)**	**Peak Area**	**% Area**
Demethylation and Ketone formation	C14H17N3O2	260.139	0.1	0.74	5.26E + 04	0.7
Demethylation and Ketone formation	C14H17N3O2	260.139	−0.1	0.93	5.71E + 04	0.7
Demethylation and Ketone formation	C14H17N3O2	260.14	1.6	1.25	3.26E + 05	4.1
Di-Oxidation and Ketone Formation	C15H19N3O4	306.145	−0.1	1.31	3.19E + 03	0
Loss of CH2 + Oxidation	C14H19N3O2	262.155	−1	1.33	2.64E + 04	0.3
Loss of C5H12N2 + Di-Oxidation and Ketone Formation	C10H7NO4	206.045	−1.1	1.45	1.48E + 04	0.2
Oxidation	C15H21N3O2	276.171	0.6	1.96	8.10E + 04	1
Loss of CH2	C14H19N3O	246.16	−0.8	2.14	1.50E + 04	0.2
Oxidation	C15H21N3O2	276.171	2.6	2.17	4.40E + 05	5.5
Loss of CH2	C14H19N3O	246.161	1.6	2.52	1.46E + 05	1.8
Oxidation	C15H21N3O2	276.171	0.4	3.51	1.56E + 04	0.2
Oxidation & Ketone	C15H19N3O3	290.15	1.3	3.66	1.91E + 04	0.2
Oxidation	C15H21N3O2	276.171	1.4	3.68	7.90E + 04	1
Oxidation & Ketone	C15H19N3O3	290.151	2.5	3.85	2.27E + 03	0
Oxidation	C15H21N3O2	276.171	1	3.97	4.60E + 03	0.1
Oxidative Deamination to Ketone	C15H18N2O2	259.144	0.4	4.2	6.08E + 03	0.1
Oxidation	C15H21N3O2	276.171	1.3	4.21	6.32E + 04	0.8
Oxidative Deamination to Alcohol	C15H20N2O2	261.16	0.1	7.14	6.85E + 04	0.9
**Putative Metabolite (MAO-A)**	**Formula**	***m/z***	**ppm**	**rt (min)**	**Peak Area**	**% Area**
Loss of NH + Desaturation	C15H18N2O	243.15	1.5	4.2	4.15E + 03	0
Oxidative Deamination to Alcohol	C15C20N2O2	261.16	2.3	7.14	1.62E + 06	14.2

**Table 4:** **Putative metabolites observed using accurate mass after two-hour incubation with pooled human hepatocytes with retention times (rt), observed*m/z*and transitions**

**Putative Metabolite (HH)**	**Formula**	***m/z***	**ppm**	**rt (min)**	**Peak Area**	**% Area**
Loss of C5H11N and CH2	C9H8N2O	161.0704	−3.2	1.36	9.02E + 03	0.1
Loss of CH2+ Gluc. Conj.	C20H27N3O7	422.1910	−2.9	1.94	3.87E + 03	0.0
Oxidation*	**C15H21N3O2**	**276.1706**	**−0.2**	**2.18**	**1.39E + 04**	**0.2**
Loss of CH2*	**C14H19N3O2**	**246.1603**	**0.8**	**2.52**	**4.06E + 04**	**0.5**
Ketone Formation	C15H19N3O2	274.1552	0.9	2.64	5.30E + 03	0.1
Oxidation*	**C15H21N3O2**	**276.1705**	**−0.5**	**3.68**	**8.42E + 03**	**0.1**
Oxidation*	**C15H21N3O2**	**276.1705**	**−0.5**	**4.22**	**1.45E + 04**	**0.2**
Loss of NH + Oxidation and Gluc. Conj.	C21H28N2O8	437.1916	−0.5	6.21	5.21E + 03	0.1
Carbamoyl Glucoronide	C22H29N3O9	480.1971	−1.1	6.40	3.72E + 04	0.4
Oxidative Deamination to Acid	C15H18N2O3	275.1402	4.3	7.14	6.65E + 05	7.4
Oxidative Deamination to Alcohol***	**C15H20N2O2**	**261.1600**	**0.8**	**7.14**	**3.01E + 04**	**0.3**

Metabolites observed after two-hour incubation with pooled human hepatocytes with retention times (rt), observed *m/z*, ppm deviance from expected mass defect, peak area, and % total chromatographic area. Bolded metabolites can be accounted for as arising from either 2D6 * or MAO-A** metabolism, or were observed in both ***

## Discussion

Isoenzyme activity screening and steady-state kinetic data suggest that CYPs 3A4, 2D6, 2C19, and also MAO-A all play a role in PQ metabolism. However, using the RAF-weighted, steady-state kinetics approach to analyse the data [11], MAO-A appears to be the predominant enzyme involved in Phase I PQ metabolism, followed by 2C19, 3A4, and 2D6, respectively. Brossi *et al* proposed a role for MAOs in the formation of the carboxy (CPQ) metabolite, and demonstrated differential K_i_ values for (+)-PQ and (−)-PQ [12]. Further, they directly demonstrated CPQ formation in liver fractions containing only MAOs. These observations appear to support the findings of the present study, indicating a major role for MAO-A, in particular, in PQ metabolism.

While 3A4 and 2C19 are commonly involved in the metabolism of anti-malarial compounds [13], neither MAO-A nor 2D6 are generally considered to be significant contributors to anti-malarial drug metabolism. However, in this study, metabolites directly observed in MAO-A and 2D6 incubations accounted for more than 93% of the total metabolite peak area observed after incubation with hepatocytes. It is also interesting to note that several anti-malarials are known inhibitors of CYP 2D6, including chloroquine (CQ), quinine, and quinidine [13,14].

Many of the metabolites identified here have been observed during the decades of research into the metabolism of PQ [15-18]. Key new findings of the present study are the attribution of many of these metabolites to production from specific CYP or MAO mediated pathways. For example, while 3A4 and 2C19 cannot be definitively eliminated from consideration as the source of PQ’s haemolytic and/or therapeutic metabolites, this study clearly demonstrates that 2D6 has a much greater intrinsic affinity for the metabolism of PQ and, at equal concentrations, produces a significantly higher amount of phenolic metabolites than either of those isoforms. The nature of the metabolites formed, including phenolic metabolites of the type first identified by Idowu *et al.*, by CYP 2D6 is highly suggestive that 2D6 may account for the well documented haemolytic effects of the drug and its efficacy, as several of the phenolic metabolites observed have been shown to re-dox cycle in the presence of NADPH and/or oxidoreductase [5,16]. This is believed to be the first time that these metabolites have been directly attributed to one single CYP pathway. If this is the case, then it is unlikely that haemolytic toxicity could be separated from efficacy through medicinal chemistry efforts aimed at avoiding CYP 2D6 metabolism, as the metabolite(s) responsible for each effect are generated in the same pathway. This has important implications for the efficacy of PQ as a treatment against relapsing malaria in 2D6 polymorphs of the poor or intermediate metabolizer phenotype, as prevalence of the poor metabolizer phenotypes are noted to be as high as 21% in some populations (German Caucasians) and the intermediate metabolizer phenotypes are estimated as high as 50% in others (Asians) [19]. Further, haemolytic toxicity could be exacerbated in extensive metabolizers. The formation of the alcohol and ring-closed form of the aldehyde by MAO-A, suggests that this enzyme acts to catalyze the first step in the pathway leading to the formation of CPQ, the major metabolite of PQ found in plasma, and ultimately drug clearance via the acyl glucoronide. It should be noted however that 2D6 produced the alcohol to a much lower level. This provides evidence that carboxy primaquine production may be mediated by both MAO-A and to a lesser extent by CYP 2D6. Further investigation in this area is needed to determine the effects of common CYP 2D6 and MAO-A inhibitors and inducers on PQ’s efficacy and toxicity.

## Abbreviations

PQ, Primaquine; CYP, Cytochrome P450; MAO, Monoamine oxidase; RAF, Relative activity factor; CPQ, Carboxy primaquine.

## Competing interests

The authors declare that they have no competing interests.

## Authors’ contributions

BSP and JCS: manuscript preparation, experimental design, data analysis and interpretation; JF, REC and CV: mass spectrometry; RB: enzyme kinetics; RJS and GAR: data interpretation, figure preparation; XJ: experimental design, data analysis and interpretation; MPK: funding, experimental design, manuscript preparation; LAW, CO and VM: funding, experimental design. Each author read and approved the final version of this manuscript.

## References

[B1] WHO World Malaria Report 2011 2011 World Health Organization, Geneva

[B2] Guerra CA Snow RW Hay SI Mapping the global extent of malaria in 2005 Trends Parasitol 2006 22 353 358 10.1016/j.pt.2006.06.006 16798089 PMC3111076

[B3] Price RN Tjitra E Guerra CA Yeung S White NJ Anstey NM Vivax malaria: neglected and not benign AmJTrop Med Hyg 2007 77 79 87 PMC2653940 18165478

[B4] Bolchoz LJ Budinsky RA McMillan DC Jollow DJ Primaquine-induced hemolytic anemia: formation and hemotoxicity of the arylhydroxylamine metabolite 6-methoxy-8-hydroxylaminoquinoline J Pharmacol Exp Ther 2001 297 509 515 11303037

[B5] Vasquez-Vivar J Augusto O Hydroxylated metabolites of the antimalarial drug primaquine JBC 1992 267 6848 6854 1313024

[B6] Beutler E Drug-induced hemolytic anemia Pharmacol Rev 1969 21 73 103 4887725

[B7] Bowman ZS Oatis JE Whelan JL Jollow DJ McMillan DC Primaquine induced hemolytic anemia: susceptibility of normal versus glutathione-depleted rat erythrocytes to 5-hydroxyprimaquine J Pharmacol Exp Ther 2004 309 79 85 10.1124/jpet.103.062984 14724225

[B8] Link CM Theoharides AD Anders JC Chung H Canfield CJ Structure-activity relationships of putative primaquine metabolites causing methemoglobin formation in canine hemolysates Toxicol Appl Pharmacol 1985 81 192 202 10.1016/0041-008X(85)90155-3 4060148

[B9] Ganesan S Tekwani BL Sahu R Tripathi LM Walker LA Cytochrome P450-dependent toxic effects of primaquine on human erythrocytes Tox App Pharm 2009 241 14 22 10.1016/j.taap.2009.07.012 19616568

[B10] Constantino L Paixao P Moreira R Portela MJ Do Rosario VE Iley J Metabolism of primaquine by liver homogenate fractions: Evidence for monoamine oxidase and cytochrome P450 involvement in the oxidative deamination of primaquine to carboxyprimaquine Exp Toxic Pathol 1999 51 299 303 10.1016/S0940-2993(99)80010-4 10445386

[B11] Crespi CL Miller VP The use of heterologously expressed drug metabolizing enzymes - state of the art and prospects for the future Pharmacol Ther 1999 84 121 131 10.1016/S0163-7258(99)00028-5 10596902

[B12] Brossi A Millet P Landau I Bembenek ME Abell CW Antimalarial activity and inhibition of monoamine oxidases A and B by exo-erythrocytic antimalarials FEBS 1987 214 291 294 10.1016/0014-5793(87)80072-8 3569526

[B13] Gia PT deVries PJ Pharmacokinetic interactions of antimalarial agents Clin Pharmacokinet 2001 40 343 373 10.2165/00003088-200140050-00003 11432537

[B14] Projean D Baune B Farinotti R Flinois J Beaune P Taburet A Ducharme J In vitro metabolism of choroquine: identification of CYP2C8, CYP3A4, and CYP2D6 as the main isoforms catalyzing n-desethylchloroquine formation Drug Metab Dispos 2003 31 748 754 10.1124/dmd.31.6.748 12756207

[B15] Baker JK McChesney JD Hufford CD Clark AM High-performance liquid chromatographic analysis of the metabolism of primaquine and the identification of a new mammalian metabolite J Chromatogr 1982 230 69 77 10.1016/S0378-4347(00)81431-0 7107769

[B16] Idowu OR Peggins JO Brewer TG Side-chain hydroxylation in the metabolism of 8-aminoquinoline antiparasitic agents Drug Metab Dispos 1995 23 18 27 7720521

[B17] Mihaly GW Ward SA Edwards G Nicholl DD Orme ML Breckenridge AM Pharmacokinetics of primaquine in man. I. Studies of the absolute bioavailability and effects of dose size Br J Clin Pharmacol 1985 19 745 750 10.1111/j.1365-2125.1985.tb02709.x 4027117 PMC1463857

[B18] Mihaly GW Ward SA Edwards G Orme ML Breckenridge AM Pharmacokinetics of primaquine in man: identification of the carboxylic acid derivative as a major plasma metabolite Br J Clin Pharmacol 1984 17 441 446 10.1111/j.1365-2125.1984.tb02369.x 6721990 PMC1463409

[B19] Neafsey P Ginsberg G Hattis D Sonawane B Genetic polymorphism in cytochrome P450 2D6 (CYP2D6): population distribution of CYP2D6 activity J Tox Env Health B 2009 12 334 361 10.1080/10937400903158342 20183526

